# Artificial Intelligence Needs Data: Challenges Accessing Italian Databases to Train AI

**DOI:** 10.1007/s41649-024-00282-9

**Published:** 2024-06-13

**Authors:** Ciara Staunton, Roberta Biasiotto, Katharina Tschigg, Deborah Mascalzoni

**Affiliations:** 1 Institute for Biomedicine, Eurac Research, Bolzano, Italy; 2 School of Law, University of KwaZulu-Natal, Durban, South Africa; 3 Department of Biomedical, Metabolic and Neural Sciences, University of Modena and Reggio Emilia, Modena, Italy; 4 Center for Research Ethics and Bioethics, Department of Public Health and Caring Sciences, Uppsala University, Uppsala, Sweden

**Keywords:** Artificial intelligence, Biobanks, Medical records, Italy, European Health Data Space

## Abstract

Population biobanks are an increasingly important infrastructure to support research and will be a much-needed resource in the delivery of personalised medicine. Artificial intelligence (AI) systems can process and cross-link very large amounts of data quickly and be used not only for improving research power but also for helping with complex diagnosis and prediction of diseases based on health profiles. AI, therefore, potentially has a critical role to play in personalised medicine, and biobanks can provide a lot of the necessary baseline data related to healthy populations that will enable the development of AI tools. To develop these tools, access to personal data, and in particular, sensitive data, is required. Such data could be accessed from biobanks. Biobanks are a valuable resource for research but accessing and using the data contained within such biobanks raise a host of legal, ethical, and social issues (ELSI). This includes the appropriate consent to manage the collection, storage, use, and sharing of samples and data, and appropriate governance models that provide oversight of secondary use of samples and data. Biobanks have developed new consent models and governance tools to enable access that address some of these ELSI-related issues. In this paper, we consider whether such governance frameworks can enable access to biobank data to develop AI. As Italy has one of the most restrictive regulatory frameworks on the use of genetic data in Europe, we examine the regulatory framework in Italy. We also look at the proposed changes under the European Health Data Space (EHDS). We conclude by arguing that currently, regulatory frameworks are misaligned and unless addressed, accessing data within Italian biobanks to train AI will be severely limited.

## Introduction

At times hailed as a ‘game-changer’, artificial intelligence (AI) has the potential to transform the provision of healthcare, through increased personalised care, improved treatment and diagnosis, and increased efficiencies in the health system (Bocas 2022). For the individual, it is anticipated that AI will have an important role to play in improving diagnostics and precision medicine, with decisions being made based on comprehensive data that includes genomics, medical history, and lifestyle data (Manne and Kantheti 2021); Hurvitz et al. 2021). On a population level, AI has the ability to analyse large datasets that include health records, images, clinical trial data, and population level data and thus enable the identification of patterns and trends (Lavigne et al. 2019).

This ‘game-changer’ for some, however, raises serious ethical and legal concerns for others. AI has the ability to collect, process, and analyse large quantities of data and this raises considerable privacy concerns (Manne and Kantheti 2021). In particular, we note and raise three concerns in the context of healthcare: first, an individual may not have consented to the collection of their data for processing by an AI; second, an individual may not be aware that an AI system is processing their data; third, unless checks are in place, it is quite possible that the personal data will be used for a purpose that an individual did not intend or expect. Related to these privacy issues are transparency and in particular the lack of transparency of many AI systems. The opacity embedded in many AI tools means that it is often not possible to explain why an AI tool has made a particular decision or recommended certain interventions (Ursin et al. 2022; Sand et al. 2022). For AI generally, this will make it harder to question or contest decisions based on AI.

In the healthcare sector, the ‘black box’ effect could limit the doctor’s ability to provide clear information to the patient on the basis for their treatment decision (Laï et al. 2020). If it is the AI tool that is making the decision, this may upend the doctor–patient relationship and the fiduciary duties that flow from this relationship. Related to this are questions of accountability and liability. The use of AI in the healthcare context involves doctors, healthcare facilities, tech developers, and at times regulatory bodies. However, who will be liable due to an incorrect decision of the AI: will it be the doctor, the healthcare provider as employer, or the tech developer? Even more problematic: can a doctor be found negligent if based on their professional judgment they do not follow a decision of an AI?

Much of these issues of liability will come down to whether the use of AI in a specific context becomes the standard of care. Before AI can be considered standard of care, however, bias in AI systems will need to be addressed (Celi et al. 2022; Guo et al. 2021; Andaur Navarro et al. 2021). This is in part due to the issues relating to diversity and representativeness of global datasets, but could also be due to bias in the scientific design and collection of a study (Fatumo et al. 2022). AI can only be representative of the datasets that it is trained on. Thus, if an AI is trained on biobank datasets that are generally representative of Caucasian populations, the AI can only be applicable to those populations. A recent example is a review reporting that clinical AI research heavily relies on datasets from the USA and China (Celi et al. 2022) meaning that most of the clinical AI applications that are trained on this data will be applicable to populations from the USA and China only. The AI applications cannot be generalizable, otherwise the tool will result in high error rates (Berisha et al. 2021). This will limit the impact of AI in healthcare, but it will further widen the healthcare divide leading to more inequity and inequality. A final issue related to bias is that there may also be bias within the data itself. For example, due to socio-economic factors, some populations may not be willing or able to engage with the healthcare sector, or there may be inequality already imbedded in the data itself. This issue of bias is important when considering restricting access to datasets, particularly from underrepresented populations.

A final point to note is the wider issue of, as with all new technologies, the danger of exaggerated or overhyped claims on the benefits of AI. Privacy International (2022) has cautioned against ‘techno-optimism’, or the belief that AI will resolve and improve many of the structural, economic, and social issues pervasive in our health systems. It has also criticised the UN High Commissioner for Human Rights Report on the Right to Privacy and AI for not challenging the assumption that AI leads to more efficient healthcare despite no evidence to support this. While AI can in theory support the delivery of a more efficient and effective healthcare system, it is unlikely to address systemic and structural problems that are inherent in many healthcare sectors.

Despite these concerns, the use of AI tools in the health sector is increasing and likely to continue to do so. It is thus incumbent upon all involved in the development of AI (that includes those involved in collection of data, governance and oversight of the data, and the development of the technology and those involved in the governance and oversight of the technology) that the development and use of AI are legally, ethically, and socially acceptable. The regulation of AI and AI ethics is very much on the political agenda with high-level AI ethics principles being developed by many organisations. This is perhaps reflective in the growing global interest in AI ethics. However, it has been cautioned that there is a lack of guidance for the practical implementation of these principles (Kargl et al. 2022) and there is divergence in many of the proposed solutions to address ethical challenges (Jobin et al. 2019).

The focus of our paper is on accessing the data to develop and train AI, particularly the impact that the governance of data access may have on the AI. Biobanks are an important resource for research generally and could be a potential data source for AI (Kozlakidis 2020). Biobanks also have clear and transparent governance procedures in place due to, in part, the ELSI concerns that arose and continue to be discussed on the appropriate collection, use, and re-use of their samples and data (Astrin and Betsou 2016; Fortin et al. 2011; Forzano et al. 2021; Hansson 2004; Gille et al. 2020; Kaye 2006). In response to some of these challenges, new consent models and new governance frameworks have been established to enable access to these samples and data for data-driven research methods (Sheehan 2011; Kaye et al. 2015; Mascalzoni et al. 2022; Cheah and Piasecki 2020). Ongoing ELSI issues persist, but biobanks could be a source of large quantities of good quality datasets. Thus, if correctly constituted, biobanks will have clear and transparent policies in place to facilitate access to their samples and data and this could include for AI.

The question we now must consider is whether the regulatory framework for biobanks currently enables access for the training of AI or if AI poses a gamechanger not only to the provision of healthcare but also to biobank regulatory frameworks. To unpack these issues, we have opted to consider these issues from an Italian perspective.

There has been some reflection on a principle-based approach to AI for Italy (Corea et al. 2023), the role that AI (specifically Chat GPT) can have in the healthcare (Vo et al. 2023) and home care (Cingolani et al. 2023) in Italy. There is limited empirical work on public attitudes towards on the use of AI in Italy generally. The empirical work on the use of AI in healthcare has tended to focus on the views of healthcare practitioners (Vo et al. 2023; Wangmo et al. 2019; Mahlknecht et al. 2023).The work on public perceptions and attitudes to sharing data for research does seem to indicate a willingness to share in specific contexts (Biasiotto et al. 2023; Viberg Johansson et al. 2021). We begin by outlining the rules on accessing personal data from Italian biobanks, particularly how the current legal landscape is blocking current research, but also the ability to use data collected for future orientated research. Next, we consider the impact that the proposed European Health Data Space (EHDS) will have on accessing data from biobanks and whether the EHDS could streamline access to Italian biobank data for AI. Finally, we discuss the uncertain and incoherent place that Italian biobanks will be in if the issues outlined are not addressed, reflecting on the impact this will have for the applicability of AI in Italy.

## Accessing Data from Biobanks in Italy

Italy has a considerable number of biobanks that include formally established biobanks, interconnected biobanks, and more ad hoc biobanks (Penasa and Tomasi 2021). Similar to other countries, Italy does not have a Biobank Act. The regulation of biobanks in Italy is best described as polycentric, with differing laws, guidance, and policies affecting the governance of biobank. Informed consent to the collection of samples and data and research ethics committee (REC) approval are required prior to the collection of samples and for the secondary use of samples. As with other jurisdictions in Europe, the General Data Protection Regulation (GDPR) has impacted the processing of personal data for biobanks in Italy. In the context of research, the GDPR was implemented in Italy through the provision on the processing of special categories of data. This provision regulates the use of health and genetic data for research and care but rather uniquely, it applies the rules on the processing of personal to samples.^1^ Thus, the rules on the processing of personal data apply to both personal data and biological samples.

Generally, research is not permitted to be carried out on health and genetic data unless certain conditions as provided for under this provision are met. First, the authorization to conduct research is limited to universities, research bodies, institutes, scientific societies, and researchers working within these organisations, operators of health professions and health bodies, individuals, public or private bodies specifically responsible for processing such as researchers, contract research organisations, laboratory analysists, etc. Second, for genetic data, only individuals, research bodies or institutions, associations, and public and private bodies that have a research purpose aimed at protecting the health of the data subject, third parties, or the community in the medical, biomedical, or genetic field can process genetic data for research. There is therefore no distinction between public and private bodies getting access to the data. Rather, the focus is on ensuring that the institution or body seeking access has a ‘research purpose’. Third, and most importantly, consent is the lawful basis for the processing of genetic data and samples in Italy.

Italy, thus, has a restrictive regulatory framework for the processing of genetic data for research in Europe, particularly when compared to the approach of the Nordics, with consent and REC required for all secondary use. Solutions to enable data’s secondary use can be found even within the confines of this restrictive regulatory environment. Biobanks like the Cooperative Health Research in South Tyrol (CHRIS) biobank have developed dynamic solutions. Since its inception in 2011, CHRIS has a governance model that is supported by REC oversights, an Access Policy implemented by the access committee, and participant consent. It has also implemented dynamic consent as its consent model. At baseline, participants have provided consent to the use of their data for certain research and data sharing. The dynamic consent platform enables participants to receive ongoing information about the use of their data, change their preferences if so desired, but also provide the study with a mechanism to get in touch with participants to ask them to consent to research not provided for at baseline (Mascalzoni et al. 2022; Biasiotto et al. 2021; Pattaro et al. 2015). Thus, through ongoing information and engagement and clear rules on data and sample access, CHRIS can conduct collaborative research involving data and sample sharing, in line with the restrictive Italian regulatory framework. Other biobanks have obtained consent for specific research areas, e.g. consent for research for metabolic conditions, based on the broad consent provisions in Recital 33 of the GDPR. In addition, the use of the samples and data in research will be subject to REC approval and other safeguards that may be introduced by a biobank to ensure that research occurs only within these specific areas.

Data-driven research methods require regulatory solutions that facilitate the use, re-use, and sharing of data for scientific research in a manner that ensures the ongoing protection of participants’ rights. Although the Italian regulatory framework may not be optimal for enabling access to the use of genetic data, researchers seeking access to data within Italian biobanks to develop AI could in theory apply for access, if it fell within the consent provided.

A decision in June 2022, however, upended governance frameworks and research processes that have been in place for Italian biobanks. In June 2022, the Italian Garante (National Supervisory Authority under the GDPR) issued an opinion in response to a request from Verona Hospital. Briefly, Verona Hospital sought to create a database for thoracic cancer to pursue research in nine specified research areas that relate to thoracic cancer. The database sought to use both retrospective and prospective data. For prospective data collection, the hospital intended to obtain the consent of prospective participants to the creation of the database and to carry out research on the samples and data in the nine specified areas. For the retrospective data it intended to process, the Hospital found that only 10% of the data subjects were contactable. Under Italian law, if informing the persons involved would render it impossible or seriously impair the aims of the research, consent is not necessary provided approval from an ethics committee and the Garante’s opinion has been obtained.

On the use of retrospective data, the Garante approved the creation of the biobank and stated that specifying the nine specific research areas related to thoracic cancer was needed to create the database. However, the Garante stated that the description of the nine areas was insufficiently specific for future research. Thus, the database could be created, but progressive specific consent would be required for any future research use.

This means that while broad research areas would be specific enough to create a database, it would not be for future research use. Biobanks in Italy therefore cannot rely on the broad consent provisions of Recital 33 of the GDPR as future use of any samples and data would require specific consent to a specific research study. For biobanks in Italy, this means that they must go back, re-contact, and re-consent their participants for future research if the original consent is not specific enough, even if their participants have already consented to the use of their data broadly within a specific category.

This has the potential to dramatically impact the operations of biobanks in Italy. Participants will already have provided their consent to their participation in research, and this may include broad typologies of research that may now be not in line with the opinion of the Garante. For some participants, they may have selected the option for their samples and data to be used in specific research areas, but that they do not want to be re-contacted in the future. Depending on the specificity of the consent, biobanks may only be able to use their samples and data in research if they re-contact and re-consent the participants. However, this will be contrary to their original consent that specified that they did not want to be re-contacted. Overall, the opinion puts the long-term sustainability of biobanks in Italy in doubt as it limits the secondary use of Italian data. Meeting the specificity of consent as required by the Garante will require re-contact and re-consent that was not envisaged at the time of establishment of a biobank and in the drafting of its governance framework. This resource implication will mean that biobanks will only be able to share data and samples for projects that it has sufficient resources for re-contact and re-consent.

The opinion will thus likely block current and future orientated research and will have considerable implications for the use of data in developing and training AI. It is unlikely that at the time of consent, biobanks will have anticipated that its data will be used specifically for training and developing AI. Thus, any projects seeking to use data from Italian biobanks for this purpose must either have budget and resources for recontact and reconsent or the data cannot be used. If the data cannot be used, then any resulting tool cannot be used on populations in Italy.

## European Health Data Space

National efforts, of which one of our authors (DM) is involved, are ongoing to develop a national workable solution to the current situation. Until such solutions are found and implemented, Italian biobanks must grapple with the implications of the Garante’s opinion. The restrictiveness of this opinion is even more stark when one considers that it came just 1 month after the introduction of the proposed regulation for EHDS. Introduced in May 2022, the EHDS is proposing to create a legal obligation to share electronic health data, if certain conditions are met. Under the draft proposal, electronic health data is broadly defined and includes electronic health records, genetic data, and population-based health data. Unlike the GDPR which only applies to personal data, the legal framework of the draft EHDS also applies to anonymous data.

Under the draft EHDS, a data user (defined as any natural or legal person), can apply for access to the electronic health data from a data holder (defined as any natural or legal person, which is an entity or a body in the health or care sector or performing research in relation to these sectors). This electronic health data may be personal data (and come within the GDPR) or anonymous data (and thus outside of the GDPR). Access can be provided for if the use falls under one of the eight specified purposes, as described in Recital 1 ‘that would benefit the society such as research, innovation, policy-making, patient safety, personalised medicine, official statistics or regulatory activities’. The draft EHDS is also proposing a change to how decisions related to access are made. Under the GDPR, this would have been made by the data controller, but under the EHDS, it will be made by a new independent body to be established in each member state, called a Health Data Access Body (HDAB).

To request access from the HDAB, an applicant is required to provide a detailed explanation of the purpose of the data use; description of the requested data; if anonymous data cannot be made available must justify the need for pseudonymised data; (undefined) safeguards to prevent unauthorized use and the rights and interests of the data holder and natural persons; estimated time period data is required; and details on a secure processing environment. If the application includes a request for personal data, it must provide detail on how the processing complies with the GDPR. Article 44 of the draft EHDS makes the importance of data minimization and purpose limitation in the HDAB’s assessment clear. Finally, an applicant should also provide information on any applicable ethical aspects. Although undefined, one would assume this relates to national ethical requirements if the processing of the data is for research.

The draft EHDS not only makes the intention to make data available clear, but also that it should be done within certain time frames. The HDAB must make an assessment within 2 months of receiving an application, a time-limit that can be extended by 2 months for complex applications. Once an application has been approved, a data permit is issued specifying the terms and conditions of the data use. The data holder must then make the data available to the data user within 2 months through a secure processing environment (for a legal analysis of the draft EHDS, see Slokenberga 2022). Thus, the time frame from receipt of application to receiving the data could be as little as 4 months.

## Caught in the Middle: Italian Biobanks and the Draft EHDS

If the draft EHDS is passed, it would mean that anyone could apply for access to a HDAB for access to data held by biobanks in Italy to develop and train AI. This would address some of the concerns that we have raised on the current Italian regulatory framework, notably that data can be accessed, AI can be trained on Italian data, and resulting AI tools can be used by the Italian population. However, closer inspection reveals that rather than streamlining access, the EHDS could leave Italian biobanks in a tug of war between national regulations and the proposed new European structure, particularly as it relates to the role of consent in decisions on the use of samples and data.

The draft EHDS creates a legal obligation to share research if the conditions outlined are met and this decision will be made by the HDAB, but what of the conditions of consent that a participant may have provided? Can data be accessed for purposes that a participant has not consented to as required by Italian law if the proposed EHDS becomes a law? Article 33(5) of the EHDS states that if consent is required by national law, ‘health data access bodies shall rely on the obligations laid down in this Chapter to provide access to electronic health data’. This in effect means that despite consent being the lawful basis for the processing of personal data in Italy, the draft EHDS is stating that for the secondary use of electronic health data, applicants need to concern themselves only with the requirements of the EHDS and not the conditions of consent. In other words, under the proposed EHDS, samples and data can be used for purposes beyond the consent provided.

It is critical that we do not assume that a legal framework for access data for certain purposes has the social licence for this data use (Carter et al. 2015), but we must also consider the impact that this proposed legal framework may have on biobanks and their relationship with participants. Biobanks in Italy have been operating under a consent model of data access, as required by national law. Their relationship with the participants and social licence to operate is based on the premise that they will use the data only for the specific research areas they have proved. If it comes into force under the proposed format, biobank participants’ data can be used for purposes beyond that as they provided in consent. We would have concerns that this would lead to mistrust of the biobank and result in participants withdrawing their samples and data.

The draft EHDS is thus creating a conflict of law situation. In a joint opinion, the European Data Protection Board (EDPB) and the European Data Protection Supervisor (EDPS) critiqued the draft EHDS on this and many other points for non-compliance with the GDPR (EDPB and EDPS 2022). The European Parliament in its draft report on the draft EHDS recommended the introduction of an opt-out for natural persons. In this way, individuals can decide to opt-out of the use of their electronic health data for any secondary purpose that they did not want their data processed. While this may go some way towards addressing concerns raised on the impact of the draft on an individual’s right to autonomy, participants may wonder why they are being afforded the opportunity to opt out only, for a purpose that they provided no consent in the first place.

Related to this, participants may want their data to be used for certain purposes only and indeed for use by certain bodies only as it is well demonstrated that individuals have preferences on their data use, particularly when it comes to commercial bodies (Middleton et al. 2020; Middleton et al. 2016; Romano et al. 2021). While some participants want to receive ongoing information and to opt out, others may want to consent to then to receive no further contact. Under the proposed EHDS, the former is possible, but not the latter. We therefore support the proposal in the draft report on an opt-out but would recommend consideration of an opt-in also for participants to choose.

In addition to use of samples and data for purposes only that are provided in the consent, biobanks will have discussed the governance of their samples and data with participants. For example, many biobanks have developed data access committees (DACs). DACs are responsible for, among other issues, ensuring that access requests are in line with participants’ consent and that there is no risk of stigmatisation or discrimination in the proposed data use (Cheah and Piasecki 2020). Participants will have been informed about this additional oversight when providing access to their samples and data. Biobanks will have explained this governance process to biobanks and understand that access to samples and data will only be provided after the necessary checks and approval by a DAC. Under the proposed EHDS, decisions on secondary use are in the hands of the HADB. There is no scope or role for a DAC or the data collector to object to the data access, even if they have legitimate grounds. We see this as problematic for two reasons. First, the governance arrangements of a biobank and how decisions on access are to be made will have been approved by a research ethics committee (REC) and also the participant as part of their informed consent. This will be a governance process that not only be subject to ethical oversight but have the support of the participant. These decisions will now be made by a yet to be developed independent entity, who will decide on access based on rules set forth in the draft EHDS, rules that may conflict with that as provided for in the biobank protocol, but also is not the body the participant has provided their consent to making decisions on their behalf. Second, it is important at times for local oversight of samples and data so that the results of the use of data can be contexualised where necessary to avoid any unintended discrimination or stigmatisation. The draft EHDS does not provide scope for this local oversight. We would call for, at a minimum, scope for the data collector to object to the sharing of data in certain circumstances, particularly if it could result in stigmatisation and discrimination, or there are concerns with the impact the sharing could have on the relationship of trust with the participants.

Finally, while the proposed HDAB process and the creation of an obligation to share data may be legally permitted, but legal legitimacy on data use alone is not enough. There are numerous examples of national legally mandated schemes on the secondary use of data failed due to public opposition (Carter et al. 2015). The question thus becomes whose responsibility is it to engage the participants on a new regulatory process: the biobank or the HDAB? We would argue that it probably falls to both. A biobank will continue to operate even if the EHDS comes into force; however, its governance process will be directly impacted by the regulatory changes. Biobanks will need to communicate the change in governance to participants, making it clear that such changes are as a result of legislative change. Biobanks will need to make it clear that data can now be accessed for purposes beyond their consent, and an opt-out (or opt-in) provided this recommendation is adopted. This is particularly critical in the Italian context if the conflict of law issue is resolved and we are to move to the governance framework as proposed in the draft EHDS. We also argue that HDABs have a role in this process. HDABs will need to work with data holders to inform and update data subjects on these considerable changes.

## Conclusion

Data intensive research methods, reliant on the use, re-use, and sharing of data, are changing the face of scientific research. Data is particularly needed to further research on AI, but diverse datasets are critical to ensuring that AI can be universally applicable. It is not the lack of availability of certain datasets that will restrict the applicability of AI, but restrictive governance models that preclude access to certain datasets risk limiting the useability of AI in certain contexts. The opinion of the Garante increases the burden of enabling access to data from Italian biobanks, a burden that does not necessarily seem justified when one considers the additional safeguards that could be introduced to balance a broad consent process under Recital 33. It is particularly concerning when one considers the impact that a restrictive governance model will have on the use of Italian data and the impact this restricted data use will have on Italian populations. Regulators must consider the risk of sharing and the risk of not sharing. Both risks need to be considered in our data intensive world. The proposed changes under the draft EHDS would enable access to data, but the role of the participants’ consent and how the recent opinion aligns with these proposed changes are unclear.

Research methods are changing, the risks and potential benefits associated with the research are changing, and our research regulatory processes need to adapt to suit this new research paradigm. Any new regulations must be clear, consistent with existing policies, avoid the introduction of any unnecessary regulatory burden, and be rooted in the reality of research practices and processes. Solutions to facilitating data use must be found, but for now, Italian biobanks appear stuck between a rock and a hard place in this regulatory tug of war. Unless this is resolved, AI cannot be trained on Italian datasets.

## Footnotes

1 Garante per la Protezione dei Dati Personali, Provvedimento n. 146 del 5 giugno 2019 “Prescrizioni relative al trattamento di categorie particolari di dati, ai sensi dell’articolo 21, comma 1 del decreto legislativo 10 agosto 2018, n. 101”, published in the Italian Official Gazette, general issue n. 176 del 29.07.2019.
